# Tetra­kis(nitrato-κ^2^ *O*,*O*′)[*N*,*N*′-1,4-phenyl­enebis(pyridine-4-carboxamide)-κ*N* ^1^](4-{[4-(pyridine-4-carboxamido-κ*N* ^1^)phen­yl]carbamo­yl}pyridin-1-ium)neodymium(III)

**DOI:** 10.1107/S1600536812011397

**Published:** 2012-03-21

**Authors:** Yun Zhang, Jiao-Jiao Hao, Hu Zhou

**Affiliations:** a School of Material Science and Engineering, Jiangsu University of Science and Technology, Zhenjiang 212003, People’s Republic of China

## Abstract

In the title compound, [Nd(NO_3_)_4_(C_18_H_15_N_4_O_2_)(C_18_H_14_N_4_O_2_)], the Nd^III^ centre is located on a twofold axis and exhibits a ten-coordinated distorted bicapped square-anti­prismatic geometry. The pyridinium NH H atom is disordered over the two ligands. Adjacent mononuclear clusters are linked through N—H⋯O and N—H⋯N hydrogen-bonding inter­actions, generating layers in the (102) plane.

## Related literature

For general background to octa­cyano­metallate-based compounds, see: Sieklucka *et al.* (2011 ▶); Zhou *et al.* (2010 ▶); Bok *et al.* (1975 ▶). For background to *N*,*N′*-bis­(4-pyridyl­formamide)-1,4-benzene, see: Niu *et al.* (2004 ▶); Pansanel *et al.* (2006 ▶); Song *et al.* (2009 ▶). 
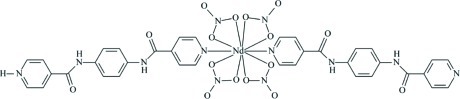


## Experimental

### 

#### Crystal data

[Nd(NO_3_)_4_(C_18_H_15_N_4_O_2_)(C_18_H_14_N_4_O_2_)]
*M* *_r_* = 1029.95
Monoclinic, 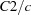

*a* = 19.856 (4) Å
*b* = 7.8491 (14) Å
*c* = 25.338 (5) Å
β = 95.153 (2)°
*V* = 3933.0 (13) Å^3^
*Z* = 4
Mo *K*α radiation
μ = 1.41 mm^−1^
*T* = 291 K
0.28 × 0.24 × 0.22 mm

#### Data collection

Bruker SMART APEX CCD diffractometer
Absorption correction: multi-scan (*SADABS*; Bruker, 2004 ▶) *T*_min_ = 0.693, *T*_max_ = 0.746
14547 measured reflections
3853 independent reflections
3510 reflections with *I* > 2σ(*I*)
*R*_int_ = 0.040

#### Refinement

*R*[*F* ^2^ > 2σ(*F* ^2^)] = 0.029
*wR*(*F* ^2^) = 0.070
*S* = 0.99
3853 reflections
294 parameters
H-atom parameters constrained
Δρ_max_ = 1.29 e Å^−3^
Δρ_min_ = −0.36 e Å^−3^

### 

Data collection: *SMART* (Bruker, 2004 ▶); cell refinement: *SAINT* (Bruker, 2004 ▶); data reduction: *SAINT*; program(s) used to solve structure: *SHELXTL* (Sheldrick, 2008 ▶); program(s) used to refine structure: *SHELXTL*; molecular graphics: *DIAMOND* (Brandenburg, 2006 ▶); software used to prepare material for publication: *SHELXTL*.

## Supplementary Material

Crystal structure: contains datablock(s) I, global. DOI: 10.1107/S1600536812011397/bt5847sup1.cif

Structure factors: contains datablock(s) I. DOI: 10.1107/S1600536812011397/bt5847Isup2.hkl

Additional supplementary materials:  crystallographic information; 3D view; checkCIF report

## Figures and Tables

**Table 1:** Hydrogen-bond geometry (Å, °)

*D*—H⋯*A*	*D*—H	H⋯*A*	*D*⋯*A*	*D*—H⋯*A*
N4—H4*A*⋯O1^i^	0.89	2.43	3.285 (4)	160
N5—H5*A*⋯O7^i^	0.89	2.23	2.966 (4)	140
N6—H6⋯N6^ii^	0.89	1.86	2.742 (5)	168

Symmetry codes: (i) 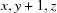; (ii) 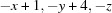.

**Fig. 1.**
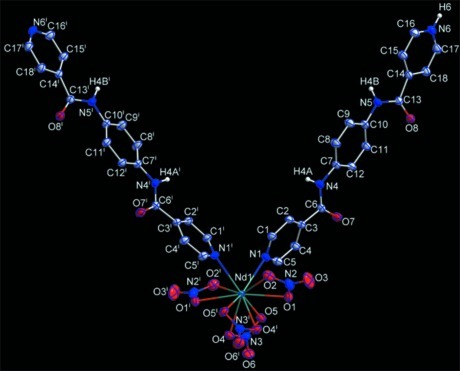
ORTEP diagram of the title compound. All hydrogen atoms bonded to carbon atoms have been omitted for clarity and thermal ellipsoids are presented at the 30% probability level. Symmetry code: (i) -x, y, -z + 1/2.

**Fig. 2.**
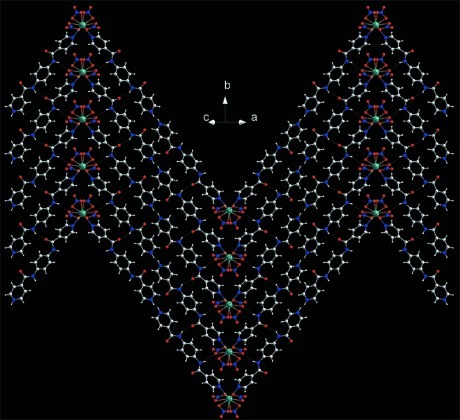
Perspective view of the title compound, showing the hydrogen-bonding interactions between adjacent mononuclear clusters.

## supplementary crystallographic information

### Comment

In the past few years, octacyanide-bearing precursors [*M*(CN)_8_]^3/4-^ (*M* = Mo, W) are frequently utilized in the construction of various dimensional structures, and the resulting materials have displayed rich magnetic properties (Sieklucka *et al.*, 2011). However, the [*M*(CN)_8_]^3/4-^-based lanthanide assemblies are relatively limited and poorly investigated, because of the liability of the lanthanide centers, the rather large anisotropic magnetic moments, and the absence of design strategies for the 4f-4 d/5 d system (Zhou *et al.*, 2010). Recently, we have used [Mo(CN)_8_]^3-^ as building block to react with Nd^3+^ and pillar ligand *N*,*N'*-bis(4-pyridylformamide)-1,4-benzene, in order to construct high-dimensional bimetallic assemblies. Unexpectedly, a new mononuclear culster, Nd(H_0.5_((*N*,*N'*-bis(4-pyridylformamide)-1,4-benzene))_2_(NO_3_)_4_, has been obtained. In the structure, the Nd^III^ ion is ten-coordinated by eight oxygen atoms of four NO_3_^-^ anions and two nitrogen atoms of two *N*,*N'*-bis(4-pyridylformamide)-1,4-benzene. The Nd—O bond lengths range from 2.513 (2) to 2.554 (2) Å, with an average value of 2.538 Å, compared to 2.671 (2) Å of Nd—N bond. Each Nd^III^ center displays a distorted bicapped square-antiprismic geometry. The first square is constructed by three oxygen atoms (O2, O4^i^ and O5; symmetry code: (i) -*x*, *y*, -*z* + 1/2) and one nitrogen atom (N1), and the second square comprises of O2^i^, O4, O5^i^, and N3^i^ atoms. The two oxygen atoms (O1 and O1^i^) occupy the two capping positions. The Nd atoms are located on a twofold axis. Thus, the adjacent mononuclear clusters are linked through the hydrogen-bonding interactions (N4—H4A···O1^ii^, N5—H5A···O7^ii^, N6—H6···N6^iii^; symmetry codes: (ii) *x*, *y* + 1, *z*; (iii) -*x +* 1, -*y* + 4, -*z*), resulting in the formation of a two layered structure.

### Experimental

Single crystals of the title compound were prepared at room temperature in the dark by slow diffusion of an acetonitrile solution (3 ml) containing Nd(NO_3_)_3_.6H_2_O (0.05 mmol) and *N*,*N'*-bis(4-pyridylformamide)-1,4-benzene (0.05 mmol) into an acetonitrile solution (15 ml) of [HN(*n*-C_4_H_9_)_3_]_3_[Mo(CN)_8_] (0.05 mmol) (Bok *et al.*, 1975). After two weeks, yellow block crystals were obtained.

### Refinement

The (C)H atoms of *N*,*N'*-bis(4-pyridylformamide)-1,4-benzene were calculated at idealized positions and included in the refinement in a riding mode. The (N)H atoms (H4A, H5A and H6) were located from difference Fourier maps and refined as riding modes with N—H = 0.89 Å and *U*(H) set to 1.2*U*_eq_(N). The H6 atom has an occupancy factor of 50% because it is disordered over two ligand molecules.

### Crystal data

**Table:**

[Nd(NO_3_)_4_(C_18_H_15_N_4_O_2_)(C_18_H_14_N_4_O_2_)]	*F*(000) = 2068
*M**_r_* = 1029.95	*D*_x_ = 1.739 Mg m^−^^3^
Monoclinic, *C*2/*c*	Mo *K*α radiation, λ = 0.71073 Å
Hall symbol: -C 2yc	Cell parameters from 5566 reflections
*a* = 19.856 (4) Å	θ = 2.5–25.6°
*b* = 7.8491 (14) Å	µ = 1.41 mm^−^^1^
*c* = 25.338 (5) Å	*T* = 291 K
β = 95.153 (2)°	Block, yellow
*V* = 3933.0 (13) Å^3^	0.28 × 0.24 × 0.22 mm
*Z* = 4	

### Data collection

**Table:**

Bruker SMART APEX CCD diffractometer	3853 independent reflections
Radiation source: fine-focus sealed tube	3510 reflections with *I* > 2σ(*I*)
Graphite monochromator	*R*_int_ = 0.040
φ and ω scans	θ_max_ = 26.0°, θ_min_ = 2.1°
Absorption correction: multi-scan (*SADABS*; Bruker, 2004)	*h* = −24→24
*T*_min_ = 0.693, *T*_max_ = 0.746	*k* = −8→9
14547 measured reflections	*l* = −31→31

### Refinement

**Table:**

Refinement on *F*^2^	Primary atom site location: structure-invariant direct methods
Least-squares matrix: full	Secondary atom site location: difference Fourier map
*R*[*F*^2^ > 2σ(*F*^2^)] = 0.029	Hydrogen site location: inferred from neighbouring sites
*wR*(*F*^2^) = 0.070	H-atom parameters constrained
*S* = 0.99	*w* = 1/[σ^2^(*F*_o_^2^) + (0.0368*P*)^2^ + 4.9413*P*] where *P* = (*F*_o_^2^ + 2*F*_c_^2^)/3
3853 reflections	(Δ/σ)_max_ < 0.001
294 parameters	Δρ_max_ = 1.29 e Å^−^^3^
0 restraints	Δρ_min_ = −0.36 e Å^−^^3^

### Special details

**Table:**

Geometry. All e.s.d.'s (except the e.s.d. in the dihedral angle between two l.s. planes) are estimated using the full covariance matrix. The cell e.s.d.'s are taken into account individually in the estimation of e.s.d.'s in distances, angles and torsion angles; correlations between e.s.d.'s in cell parameters are only used when they are defined by crystal symmetry. An approximate (isotropic) treatment of cell e.s.d.'s is used for estimating e.s.d.'s involving l.s. planes.
Refinement. Refinement of *F*^2^ against ALL reflections. The weighted *R*-factor *wR* and goodness of fit *S* are based on *F*^2^, conventional *R*-factors *R* are based on *F*, with *F* set to zero for negative *F*^2^. The threshold expression of *F*^2^ > σ(*F*^2^) is used only for calculating *R*-factors(gt) *etc*. and is not relevant to the choice of reflections for refinement. *R*-factors based on *F*^2^ are statistically about twice as large as those based on *F*, and *R*- factors based on ALL data will be even larger.

### Fractional atomic coordinates and isotropic or equivalent isotropic displacement parameters (Å^2^)

**Table:**

	*x*	*y*	*z*	*U*_iso_*/*U*_eq_	Occ. (<1)
Nd1	0.0000	0.03144 (3)	0.2500	0.02816 (8)	
O1	0.12544 (12)	−0.0024 (3)	0.23734 (10)	0.0500 (6)	
N1	0.05042 (13)	0.2942 (3)	0.19938 (10)	0.0375 (6)	
C1	0.08058 (16)	0.4270 (4)	0.22500 (13)	0.0410 (7)	
H1	0.0736	0.4421	0.2605	0.049*	
O2	0.10174 (13)	0.1272 (3)	0.30864 (9)	0.0541 (6)	
N2	0.14665 (14)	0.0697 (4)	0.28005 (13)	0.0522 (8)	
C2	0.12163 (16)	0.5434 (4)	0.20184 (12)	0.0391 (7)	
H2	0.1411	0.6339	0.2214	0.047*	
O3	0.20657 (14)	0.0853 (5)	0.29318 (15)	0.0981 (12)	
N3	−0.02515 (13)	−0.2346 (3)	0.16730 (10)	0.0386 (6)	
C3	0.13313 (14)	0.5224 (4)	0.14925 (12)	0.0345 (6)	
O4	−0.05520 (12)	−0.2346 (3)	0.20850 (9)	0.0493 (6)	
N4	0.18418 (12)	0.7980 (3)	0.13715 (10)	0.0390 (6)	
H4A	0.1615	0.8285	0.1644	0.047*	
C4	0.10281 (17)	0.3846 (4)	0.12217 (12)	0.0412 (7)	
H4	0.1097	0.3652	0.0868	0.049*	
O5	0.01619 (12)	−0.1125 (3)	0.16218 (9)	0.0497 (6)	
N5	0.32465 (13)	1.3637 (3)	0.06645 (11)	0.0425 (6)	
H5A	0.3067	1.4584	0.0785	0.051*	
C5	0.06243 (16)	0.2773 (4)	0.14848 (12)	0.0413 (7)	
H5	0.0420	0.1866	0.1296	0.050*	
O6	−0.03444 (14)	−0.3447 (4)	0.13396 (10)	0.0657 (7)	
N6	0.45861 (13)	1.8629 (3)	0.00603 (11)	0.0449 (7)	
H6	0.4815	1.9600	0.0045	0.054*	0.50
C6	0.17860 (15)	0.6371 (4)	0.11988 (12)	0.0358 (7)	
O7	0.20613 (13)	0.5820 (3)	0.08216 (10)	0.0566 (6)	
C7	0.22349 (15)	0.9311 (4)	0.11767 (13)	0.0366 (7)	
O8	0.38974 (12)	1.2586 (3)	0.00483 (10)	0.0542 (6)	
C8	0.23701 (19)	1.0708 (4)	0.15034 (14)	0.0516 (9)	
H8	0.2231	1.0708	0.1844	0.062*	
C9	0.27096 (19)	1.2096 (5)	0.13266 (14)	0.0525 (9)	
H9	0.2790	1.3035	0.1547	0.063*	
C10	0.29314 (15)	1.2111 (4)	0.08252 (12)	0.0384 (7)	
C11	0.28130 (16)	1.0710 (4)	0.05014 (13)	0.0404 (7)	
H11	0.2966	1.0703	0.0165	0.048*	
C12	0.24663 (16)	0.9308 (4)	0.06760 (13)	0.0401 (7)	
H12	0.2389	0.8366	0.0456	0.048*	
C13	0.36928 (15)	1.3780 (4)	0.02994 (12)	0.0376 (7)	
C14	0.39656 (15)	1.5559 (4)	0.02252 (12)	0.0363 (7)	
C15	0.39694 (17)	1.6835 (4)	0.05940 (13)	0.0445 (8)	
H15	0.3761	1.6679	0.0905	0.053*	
C16	0.42859 (18)	1.8357 (4)	0.04990 (14)	0.0490 (8)	
H16	0.4288	1.9219	0.0751	0.059*	
C17	0.45621 (16)	1.7421 (5)	−0.03105 (13)	0.0470 (8)	
H17	0.4757	1.7630	−0.0624	0.056*	
C18	0.42595 (16)	1.5886 (4)	−0.02424 (13)	0.0432 (7)	
H18	0.4250	1.5063	−0.0507	0.052*	

### Atomic displacement parameters (Å^2^)

**Table:**

	*U*^11^	*U*^22^	*U*^33^	*U*^12^	*U*^13^	*U*^23^
Nd1	0.03137 (12)	0.02393 (12)	0.03054 (12)	0.000	0.01028 (8)	0.000
O1	0.0389 (12)	0.0579 (16)	0.0550 (15)	0.0037 (10)	0.0140 (11)	0.0070 (11)
N1	0.0421 (14)	0.0277 (14)	0.0441 (15)	−0.0043 (11)	0.0117 (11)	0.0039 (11)
C1	0.0498 (18)	0.0317 (18)	0.0434 (17)	−0.0036 (14)	0.0149 (14)	0.0018 (13)
O2	0.0614 (16)	0.0571 (16)	0.0424 (13)	−0.0094 (12)	−0.0036 (11)	−0.0003 (11)
N2	0.0366 (15)	0.058 (2)	0.061 (2)	−0.0048 (13)	−0.0022 (14)	0.0215 (15)
C2	0.0446 (17)	0.0303 (16)	0.0434 (17)	−0.0051 (13)	0.0097 (13)	−0.0034 (13)
O3	0.0409 (16)	0.122 (3)	0.126 (3)	−0.0114 (17)	−0.0256 (17)	0.027 (2)
N3	0.0445 (15)	0.0312 (14)	0.0406 (14)	−0.0007 (11)	0.0067 (11)	−0.0052 (11)
C3	0.0329 (14)	0.0270 (15)	0.0445 (17)	0.0001 (12)	0.0085 (12)	0.0048 (12)
O4	0.0651 (15)	0.0403 (13)	0.0457 (13)	−0.0136 (11)	0.0228 (11)	−0.0057 (10)
N4	0.0403 (14)	0.0321 (15)	0.0472 (15)	−0.0051 (11)	0.0187 (11)	0.0033 (11)
C4	0.0526 (19)	0.0349 (18)	0.0373 (16)	−0.0075 (15)	0.0105 (14)	0.0009 (13)
O5	0.0597 (14)	0.0437 (14)	0.0494 (13)	−0.0146 (12)	0.0247 (11)	−0.0082 (11)
N5	0.0478 (15)	0.0272 (14)	0.0557 (16)	−0.0075 (11)	0.0226 (13)	−0.0010 (11)
C5	0.0478 (18)	0.0334 (17)	0.0430 (18)	−0.0086 (14)	0.0050 (14)	0.0015 (13)
O6	0.0768 (18)	0.0620 (18)	0.0598 (16)	−0.0183 (14)	0.0153 (13)	−0.0294 (13)
N6	0.0406 (15)	0.0378 (16)	0.0565 (17)	−0.0121 (12)	0.0053 (12)	0.0087 (13)
C6	0.0349 (15)	0.0312 (17)	0.0424 (17)	−0.0015 (12)	0.0102 (13)	0.0020 (12)
O7	0.0724 (17)	0.0382 (13)	0.0647 (16)	−0.0087 (12)	0.0373 (13)	−0.0040 (11)
C7	0.0339 (15)	0.0279 (17)	0.0495 (18)	−0.0045 (12)	0.0117 (13)	0.0043 (12)
O8	0.0538 (14)	0.0405 (14)	0.0723 (16)	−0.0109 (11)	0.0276 (12)	−0.0106 (12)
C8	0.067 (2)	0.043 (2)	0.049 (2)	−0.0161 (17)	0.0284 (17)	−0.0039 (15)
C9	0.066 (2)	0.042 (2)	0.053 (2)	−0.0170 (17)	0.0239 (17)	−0.0108 (16)
C10	0.0352 (15)	0.0317 (17)	0.0498 (18)	−0.0056 (13)	0.0123 (13)	0.0026 (13)
C11	0.0438 (17)	0.0389 (19)	0.0396 (17)	−0.0097 (14)	0.0100 (13)	0.0025 (13)
C12	0.0432 (17)	0.0326 (18)	0.0451 (18)	−0.0092 (13)	0.0081 (14)	−0.0016 (13)
C13	0.0333 (15)	0.0381 (19)	0.0422 (17)	−0.0051 (13)	0.0074 (13)	−0.0019 (14)
C14	0.0320 (15)	0.0371 (18)	0.0405 (16)	−0.0055 (12)	0.0074 (12)	0.0030 (13)
C15	0.0502 (19)	0.043 (2)	0.0416 (17)	−0.0131 (15)	0.0125 (14)	−0.0019 (14)
C16	0.053 (2)	0.0404 (19)	0.054 (2)	−0.0121 (16)	0.0068 (16)	−0.0053 (15)
C17	0.0449 (18)	0.050 (2)	0.0473 (19)	−0.0064 (16)	0.0122 (15)	0.0096 (16)
C18	0.0439 (18)	0.0451 (19)	0.0422 (17)	−0.0077 (15)	0.0122 (14)	−0.0024 (14)

### Geometric parameters (Å, º)

**Table:**

Nd1—O2	2.513 (2)	C4—H4	0.9300
Nd1—O2^i^	2.513 (2)	N5—C13	1.342 (4)
Nd1—O5	2.542 (2)	N5—C10	1.428 (4)
Nd1—O5^i^	2.542 (2)	N5—H5A	0.8900
Nd1—O4	2.542 (2)	C5—H5	0.9300
Nd1—O4^i^	2.542 (2)	N6—C16	1.325 (4)
Nd1—O1^i^	2.554 (2)	N6—C17	1.333 (4)
Nd1—O1	2.554 (2)	N6—H6	0.8900
Nd1—N1	2.671 (2)	C6—O7	1.222 (4)
Nd1—N1^i^	2.671 (2)	C7—C8	1.385 (5)
Nd1—N2^i^	2.958 (3)	C7—C12	1.388 (4)
Nd1—N2	2.958 (3)	O8—C13	1.222 (4)
O1—N2	1.260 (4)	C8—C9	1.377 (5)
N1—C1	1.340 (4)	C8—H8	0.9300
N1—C5	1.339 (4)	C9—C10	1.382 (4)
C1—C2	1.389 (4)	C9—H9	0.9300
C1—H1	0.9300	C10—C11	1.380 (4)
O2—N2	1.281 (4)	C11—C12	1.391 (4)
N2—O3	1.213 (4)	C11—H11	0.9300
C2—C3	1.382 (4)	C12—H12	0.9300
C2—H2	0.9300	C13—C14	1.516 (4)
N3—O6	1.211 (3)	C14—C15	1.369 (4)
N3—O4	1.248 (3)	C14—C18	1.391 (4)
N3—O5	1.276 (3)	C15—C16	1.381 (5)
C3—C4	1.389 (4)	C15—H15	0.9300
C3—C6	1.517 (4)	C16—H16	0.9300
N4—C6	1.338 (4)	C17—C18	1.364 (5)
N4—C7	1.419 (4)	C17—H17	0.9300
N4—H4A	0.8902	C18—H18	0.9300
C4—C5	1.376 (4)		
			
O2—Nd1—O2^i^	145.18 (12)	O3—N2—O1	121.7 (4)
O2—Nd1—O5	119.51 (8)	O3—N2—O2	121.7 (4)
O2^i^—Nd1—O5	76.91 (8)	O1—N2—O2	116.6 (3)
O2—Nd1—O5^i^	76.91 (8)	O3—N2—Nd1	179.0 (3)
O2^i^—Nd1—O5^i^	119.51 (8)	O1—N2—Nd1	59.20 (15)
O5—Nd1—O5^i^	127.20 (11)	O2—N2—Nd1	57.46 (15)
O2—Nd1—O4	141.65 (8)	C3—C2—C1	118.9 (3)
O2^i^—Nd1—O4	72.99 (8)	C3—C2—H2	120.6
O5—Nd1—O4	49.93 (7)	C1—C2—H2	120.6
O5^i^—Nd1—O4	85.01 (8)	O6—N3—O4	121.9 (3)
O2—Nd1—O4^i^	72.99 (8)	O6—N3—O5	121.6 (3)
O2^i^—Nd1—O4^i^	141.65 (8)	O4—N3—O5	116.5 (2)
O5—Nd1—O4^i^	85.01 (8)	O6—N3—Nd1	178.8 (2)
O5^i^—Nd1—O4^i^	49.93 (7)	O4—N3—Nd1	58.16 (14)
O4—Nd1—O4^i^	69.51 (11)	O5—N3—Nd1	58.30 (14)
O2—Nd1—O1^i^	134.28 (8)	C2—C3—C4	117.9 (3)
O2^i^—Nd1—O1^i^	50.51 (8)	C2—C3—C6	124.0 (3)
O5—Nd1—O1^i^	105.38 (8)	C4—C3—C6	118.1 (3)
O5^i^—Nd1—O1^i^	69.04 (8)	N3—O4—Nd1	97.18 (16)
O4—Nd1—O1^i^	65.00 (8)	C6—N4—C7	127.8 (3)
O4^i^—Nd1—O1^i^	104.58 (8)	C6—N4—H4A	118.3
O2—Nd1—O1	50.51 (8)	C7—N4—H4A	113.8
O2^i^—Nd1—O1	134.28 (8)	C5—C4—C3	118.9 (3)
O5—Nd1—O1	69.04 (8)	C5—C4—H4	120.6
O5^i^—Nd1—O1	105.38 (8)	C3—C4—H4	120.6
O4—Nd1—O1	104.58 (8)	N3—O5—Nd1	96.42 (16)
O4^i^—Nd1—O1	65.00 (8)	C13—N5—C10	126.9 (3)
O1^i^—Nd1—O1	168.06 (11)	C13—N5—H5A	118.5
O2—Nd1—N1	74.74 (8)	C10—N5—H5A	113.8
O2^i^—Nd1—N1	78.52 (8)	N1—C5—C4	124.6 (3)
O5—Nd1—N1	80.83 (8)	N1—C5—H5	117.7
O5^i^—Nd1—N1	147.80 (8)	C4—C5—H5	117.7
O4—Nd1—N1	127.02 (7)	C16—N6—C17	119.1 (3)
O4^i^—Nd1—N1	131.83 (8)	C16—N6—H6	116.3
O1^i^—Nd1—N1	123.55 (8)	C17—N6—H6	124.4
O1—Nd1—N1	66.92 (8)	O7—C6—N4	124.1 (3)
O2—Nd1—N1^i^	78.52 (8)	O7—C6—C3	120.1 (3)
O2^i^—Nd1—N1^i^	74.74 (8)	N4—C6—C3	115.8 (3)
O5—Nd1—N1^i^	147.80 (8)	C8—C7—C12	119.0 (3)
O5^i^—Nd1—N1^i^	80.83 (8)	C8—C7—N4	117.3 (3)
O4—Nd1—N1^i^	131.83 (8)	C12—C7—N4	123.7 (3)
O4^i^—Nd1—N1^i^	127.02 (7)	C9—C8—C7	120.4 (3)
O1^i^—Nd1—N1^i^	66.92 (8)	C9—C8—H8	119.8
O1—Nd1—N1^i^	123.55 (8)	C7—C8—H8	119.8
N1—Nd1—N1^i^	78.88 (11)	C10—C9—C8	120.8 (3)
O2—Nd1—N2^i^	147.38 (8)	C10—C9—H9	119.6
O2^i^—Nd1—N2^i^	25.44 (8)	C8—C9—H9	119.6
O5—Nd1—N2^i^	91.07 (8)	C9—C10—C11	119.3 (3)
O5^i^—Nd1—N2^i^	94.10 (9)	C9—C10—N5	117.0 (3)
O4—Nd1—N2^i^	66.36 (8)	C11—C10—N5	123.7 (3)
O4^i^—Nd1—N2^i^	124.59 (9)	C10—C11—C12	120.3 (3)
O1^i^—Nd1—N2^i^	25.07 (9)	C10—C11—H11	119.9
O1—Nd1—N2^i^	157.96 (8)	C12—C11—H11	119.9
N1—Nd1—N2^i^	101.58 (9)	C7—C12—C11	120.2 (3)
N1^i^—Nd1—N2^i^	69.05 (8)	C7—C12—H12	119.9
O2—Nd1—N2	25.44 (8)	C11—C12—H12	119.9
O2^i^—Nd1—N2	147.38 (8)	O8—C13—N5	124.5 (3)
O5—Nd1—N2	94.10 (9)	O8—C13—C14	120.2 (3)
O5^i^—Nd1—N2	91.07 (8)	N5—C13—C14	115.3 (3)
O4—Nd1—N2	124.59 (9)	C15—C14—C18	118.0 (3)
O4^i^—Nd1—N2	66.36 (8)	C15—C14—C13	124.7 (3)
O1^i^—Nd1—N2	157.96 (8)	C18—C14—C13	117.2 (3)
O1—Nd1—N2	25.07 (9)	C14—C15—C16	119.2 (3)
N1—Nd1—N2	69.05 (8)	C14—C15—H15	120.4
N1^i^—Nd1—N2	101.58 (9)	C16—C15—H15	120.4
N2^i^—Nd1—N2	168.35 (12)	N6—C16—C15	122.1 (3)
N2—O1—Nd1	95.73 (19)	N6—C16—H16	118.9
C1—N1—C5	115.7 (3)	C15—C16—H16	118.9
C1—N1—Nd1	122.6 (2)	N6—C17—C18	121.8 (3)
C5—N1—Nd1	119.53 (19)	N6—C17—H17	119.1
N1—C1—C2	124.1 (3)	C18—C17—H17	119.1
N1—C1—H1	118.0	C17—C18—C14	119.6 (3)
C2—C1—H1	118.0	C17—C18—H18	120.2
N2—O2—Nd1	97.10 (18)	C14—C18—H18	120.2

Symmetry code: (i) −*x*, *y*, −*z*+1/2.

### Hydrogen-bond geometry (Å, º)

**Table:**

*D*—H···*A*	*D*—H	H···*A*	*D*···*A*	*D*—H···*A*
N4—H4*A*···O1^ii^	0.89	2.43	3.285 (4)	160
N5—H5*A*···O7^ii^	0.89	2.23	2.966 (4)	140
N6—H6···N6^iii^	0.89	1.86	2.742 (5)	168

Symmetry codes: (ii) *x*, *y*+1, *z*; (iii) −*x*+1, −*y*+4, −*z*.
